# Examining the changing demand for orthotic service provision from routinely collected digital patient data: A national retrospective descriptive study across three clinics in Cambodia

**DOI:** 10.1371/journal.pone.0338461

**Published:** 2025-12-09

**Authors:** Charlotte Spurway, Alex Dickinson, Lucy Gates, Cheryl Metcalf, Sisary Kheng, Carson Harte, Bunthoeun Sam, Sam Simpson, Amos Channon

**Affiliations:** 1 Health Economics Unit, School of Health Sciences, University of Birmingham, Birmingham, United Kingdom; 2 Faculty of Engineering & Physical Sciences, University of Southampton, Southampton, United Kingdom; 3 Institute for Life Sciences, University of Southampton, Southampton, United Kingdom; 4 Exceed Research Network, Lisburn, United Kingdom; 5 Faculty of Medicine, University of Southampton, Southampton, United Kingdom; 6 Exceed Worldwide, Phnom Penh, Cambodia; 7 Exceed Worldwide, Lisburn, United Kingdom; 8 Centre for Global Health and Policy, University of Southampton, Southampton, United Kingdom; St Mary's University, UNITED STATES OF AMERICA

## Abstract

Available data on the demand for orthotic services is limited, especially in low- and middle-income countries, yet the development and delivery of services relies on this information. In this study, routinely collected digital patient records are used to provide insights into orthotic service users from three Cambodian physical rehabilitation centres. Analysis of the digital patient records from 1998–2019 investigated trends by sex, clinic, reason for orthosis use, orthotic type, and age. The analysis showed that the characteristics of service users and the provision of orthotics have changed over time. The predominant reason for orthosis use prior to 2006 was polio, whereas in 2019 it was cerebral palsy. Ankle Foot Orthoses (AFOs) are the most common type of orthosis provided; however, key differences have been found between type of orthosis and age which suggests older individuals have different experiences of physical rehabilitation compared to younger age groups. Longitudinal trends indicate a substantial reduction in orthotics appointments which may be associated with change in service delivery and changing service user characteristics. The study has illustrated the insights which can be derived from digital patient records into the demand for orthotics services over two decades and demonstrates the need for targeted resources and planning for the provision of services.

## Introduction

Assistive products that aid mobility and limb function have the potential to benefit over one billion people worldwide [1,2]. The World Health Organisation (WHO) Rehab2023 programme highlights the importance of assistive products in achieving equitable progress towards universal health coverage for people with disabilities [3,4]. Orthoses are a type of assistive product designed to support, align, or improve the function of body parts for individuals with physical impairments.

Access to orthoses can promote a person’s independence and participation in society, while also supporting individual functioning [5]. According to ATscale, investing in assistive technologies yields significant economic, health and social benefits, with every dollar invested into assistive technology providing a return of US$9 [6]. However, evidence on orthosis users and the need for orthotic devices in low-and-middle-income countries (LMICs) remains limited. Globally, the demand for orthotic devices is unknown, especially in comparison to prosthetics [7], which are often delivered alongside orthoses in physical rehabilitation or Prosthetic and Orthotic (P&O) services.

Orthotics are custom-made medical devices that provide both short-term protective and corrective benefits, as well as long-term support for individuals with a range of conditions, such as early developmental disorders (i.e., cerebral palsy and clubfoot), polio, stroke and spinal cord injuries [8]. Unlike a one-time intervention, orthotics require ongoing follow-up and adjustments as the patient’s body changes (e.g., during childhood growth or recovery from injury), or when ill-fitting devices lead to skin problems, pain, or reduced mobility [8]. This means long-term patient records and consistent follow-up are just as important as the initial fitting.

Access to orthotic services in LMICs is challenging for users, leading to inequities in the provision of assistive products between population groups. Furthermore, there is a lack of awareness of the need for orthotic services amongst policy makers, low political prioritisation to provide assistive products, limited investment, and demand- and supply-side barriers [1,9].

Routinely collected digital patient data can play an important role in overcoming these access challenges by providing an evidence base to inform service planning and promote equitable provision [10]. Beyond improving access, such data are also important for maintaining continuity of care over time, supporting the long-term monitoring and follow-up that orthosis users require. By capturing data on who is accessing services, and which orthotic devices are most in demand, routine data can help stakeholders make informed decisions for underserved populations. Previous research has highlighted the benefits of a strong routine collection of this data and how this can be improved [11] and links with guidelines for the prescription of orthotic services and device delivery [8]. Analysing this digital health data is essential for making operational and strategic decisions in health systems [12]. For example, understanding the demographics of service users could support patient-centred care and support more efficient resource allocation. However, in Cambodia and many other LMICs, a lack of comprehensive data on orthotic services impacts efforts to plan effectively for long-term population needs.

In Cambodia, physical rehabilitation and assistive products, including devices such as orthotics and mobility aids are not widely available within the public healthcare system, and government funding is limited [13]. There are currently 11 physical rehabilitation centres which provide P&O services free of charge operating in Cambodia. Six are run and primarily funded by international organisations, including Exceed Worldwide (Exceed), the International Committee of the Red Cross (ICRC), and Humanity & Inclusion, while the remaining five are fully operated and funded by the government agency, the People with Disability Foundation [14]. Reductions in donor funding have significantly impacted services, with the government providing some additional funding to support organisations and outreach activities [15].

This is a convenience study drawing on administrative patient data from the three Exceed-managed centres, which were accessible through the study partnership. The remaining eight centres, operated by ICRC, Humanity & Inclusion, or the People with Disability Foundation, were not included, however, they provide similar services under different management. Given the lack of prevalence data or large-scale surveys on orthotic users, administrative data from P&O services can be used to examine service demand and provide evidence to inform service delivery and practices. Ultimately, this could help ensure equitable rehabilitation access across different populations. This study aims to analyse patterns of P&O centre access for orthotic device users in Cambodia, with the following objectives:

i) To understand the profile of orthotic service users at three physical rehabilitation P&O centres in Cambodia, and
ii) To explore how orthotic service provision and use has changed over time.

This paper seeks to address this knowledge gap and contribute to the limited evidence on orthoses in Cambodia, an LMIC in Southeast Asia.

## Materials and methods

Building upon the methodology of a prior study on prosthetic limbs [16], this study analysed data collected at three physical rehabilitation P&O centres in Phnom Penh, Kampong Chhnang and Sihanoukville/ Kampong Som, Cambodia, as part of routine care. The previous approach used for prosthetics demonstrated feasibility for analysing patient data in the Cambodian context, and using a consistent methodology here allows for some degree of comparability between P&O service use. These centres are operated by Exceed which uses the PMS-5 digital database management system created by ICRC (Geneva, Switzerland). Exceed operates three of the 11 P&O clinics in Cambodia, delivering orthotic services to people living in the respective provinces and neighbouring areas. Individuals may be referred to the service by a healthcare provider, however for orthotics, referrals are typically made through Exceed’s community outreach services or via self-referral.

Ethical approval for the study was provided by the University of Southampton institutional ethics committee (ERGO ID:51898) and Cambodian National Ethics Committee for Health Research (230&311NECHR). The raw data was extracted from the PMS-5 database into Excel format on 05-12-2019 and 22-07-2020. It was anonymised and all personal details (e.g., patient name, address, contact details) were removed by one of the co-authors based at the Phnom Penh clinic (BS) before being transferred to the rest of the author team. The anonymised data was then converted from Excel format for analysis in Stata V16. The dataset includes information about service user demographics and their consultations.

In the data, each row represents a visit to the clinic by an individual, with appointments recorded as either an assessment, delivery or repair, meaning clients can have multiple rows of data depending on the number and type of appointments. All appointments recorded from 1998 (when electronic records were first introduced) until the end of 2019 were included in the analysis. The variables included in this study are sex, clinic, reason for using an orthosis, type of orthosis prescribed, age at time of consultation, age at first appointment and age at the end of 2019. Some records had missing data for certain variables. Where there was missing data, this has been noted in the results tables.

For the analysis, a subset of clients was identified as ‘active’ if they had at least one appointment within the last seven years of the study period (since 1^st^ January 2013), as recommended by the Exceed Cambodia Country Director (SK). This timeframe was selected because clients remain active in the Exceed data management system for seven years following their most recent service use, those who have not accessed the service within this period are considered inactive. ‘Active’ clients were used to examine current trends, while the full dataset (‘all’ clients) was used to assess changes over time. This is a descriptive study to provide basic information about orthosis users, hence only descriptive statistics were used in the analysis. Chi-square tests were performed to assess differences between variables.

## Results

**Fig 1:**
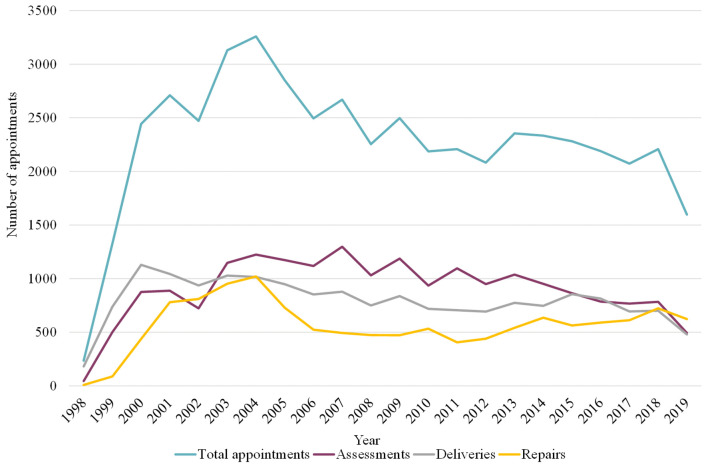
Total number of appointments for orthotic devices for all clients from 1998 to 2019. Total appointments include the total number of assessments, deliveries and repairs.

A total of 49,861 appointments were completed from 1998**–**2019, involving 12,170 individual clients, of whom 4,000 were classified as active. The total number of appointments (assessments, deliveries, and repairs) peaked in 2004 but has declined since 2005 (Fig 1) (S1 Table in S1 File). From 2004**–**2006, repairs declined more rapidly than other appointment types, however, total repairs increased gradually after 2006 to become the most common type of appointment in 2019, surpassing assessments (Fig 1). The total number of orthoses delivered peaked in 2000 and has steadily declined since. A notable decline across all three appointment types is evident in 2018**–**2019 (Fig 1).

### Client demographics

Table 1 presents the demographics of all and active clients, though this section focuses on active users to show current service users. Overall, the percentage of female active clients is nearly 10% lower than male active clients. The reasons for orthosis use vary, with cerebral palsy being the most common among active clients (26.9%), other (15.9%), polio (11.7%), followed by paralysis (8.3%) (Table 1). At their first appointment, 42.0% of active clients were aged 0–8 years, and a further 18.6% were aged 9–17 years. In total, this means that 60% of active clients were under 18 at the time of their first appointment. By contrast, the proportion of clients aged 18–49 years at their first appointment was comparatively low. The proportion then rose again among clients aged 50 and over, reaching 17.1% (Table 1).

**Table 1:** Number and percentage of all and active clients by demographic characteristics among service users from 1998 to 2019.

		All Clients (%), N = 12170	Count	Active clients (%), N = 4248	Count
Sex	Female	44.8	5447	45.3	1926
	Male	55.2	6723	54.7	2322
Clinic	Phnom Penh	51.6	6278	45.6	1936
	Kampong Chhnang	29.0	3534	34.3	1456
	Sihanoukville	19.4	2358	20.2	856
Reason for using orthosis	Cerebral Palsy	20.1	2440	26.9	1144
	Other^a^	15.8	1923	15.9	677
	Polio	15.7	1875	11.7	498
	Paralysis	10.8	1311	8.3	354
	Dislocation/fracture	8.7	1053	6.1	261
	Stroke	2.8	344	5.8	245
	Clubfoot	6.5	796	5.7	242
	Infection/disease	5.7	689	4.6	197
	Short leg	2.2	268	4.2	178
	Scoliosis (AIS)	2.5	302	3.7	156
	Missing	3.2	385	2.9	123
	Congenital	3.4	418	2.5	106
	Trauma/injury	3.0	364	1.6	67
Age at first appointment (years)	0-8	34.1	4153	42.0	1784
	9-17	21.8	2658	18.6	792
	18-29	13.8	1674	9.4	397
	30-39	7.7	938	7.0	299
	40-49	7.1	866	5.9	251
	50+	15.5	1882	17.1	725
Age at the end of 2019 (years)^b^	0-8	–	–	25.3	1073
	9-17	–	–	21.9	932
	18-29	–	–	15.0	637
	30-39	–	–	11.1	473
	40-49	–	–	5.4	231
	50+	–	–	21.2	902

^a^Other All clients: Other = 926, Contracture = 246, Torticollis = 200, Unknown = 198, Bowleg = 85, Pain = 54, Visual impairment = 40, Hearing impairment = 39, Valgus = 39, Malnutrition = 31, Equinus = 26, Flat foot = 25 and Varus = 25

^b^Age at the end of 2019 was calculated only for active clients, as some individuals in the full dataset would be over 100 years old and are likely deceased.

Significant differences were observed between sexes in the reasons for orthosis use (P < 0.01; Table 2). Among active clients with cerebral palsy, polio, or other conditions, only small differences were found between males and females (range 0.9–1.6%) (Table 2). However, notable differences were found for some reasons for orthosis use, including 6.3% of active female clients used an orthosis for adolescent idiopathic scoliosis (AIS), compared to only 1.5% of male clients (Table 2).

**Table 2:** Number and percentage of active clients by reason for orthosis use and sex among service users from 1998 to 2019.

		Active clients (%)		Count n = 4248	
		F	M	F	M
Reason for orthosis use^a^	Cerebral Palsy	27.8	26.2	536	608
	Other^b^	16.5	15.5	317	360
	Polio	11.2	12.1	216	282
	Paralysis	5.8	10.5	111	243
	Dislocation/fracture	6.6	5.8	127	134
	Stroke	5.1	6.3	98	147
	Clubfoot	4.8	6.4	94	148
	Infection/disease	5.2	4.2	100	97
	Short leg	4.2	4.2	81	97
	Scoliosis (AIS)	6.3	1.5	122	34
	Missing	2.6	3.1	50	73
	Congenital	2.6	2.4	50	56
	Trauma/injury	1.3	1.9	24	43
**Total**		**100.0**	**100.0**	**1939**	**2339**

^a^P value (chi-square test) significant at 1% level (P < 0.01)

^b^Other active clients: Other = 263, Torticollis = 129, Contracture = 96, Unknown = 75, Bowleg = 42, Valgus/Varus = 25, Pain = 23, Flat foot = 12, Malnutrition = 6, and Equinus = 6.

### Types of orthoses prescribed

AFOs comprised over 40% of the devices delivered (7762/17,523 total devices), followed by KAFOs (at 24%, 4204/17,523 total devices). This means that AFOs and KAFOs account for over two thirds of all orthoses delivered since 1998 (Table 3). In addition, shoe raises (SRs) comprised over 11% orthotic deliveries (2002/17,523 total devices). Smaller but substantial numbers (over 600 devices each) of FOs, SFABs for club foot, upper limb (including wrist and hand orthoses), and spinal orthoses were also provided.

**Table 3:** Number and percentage of orthoses provided to all clients by type among service users from 1998 to 2019.

		Percent (%) N^a^ = 17523	Count
Type of orthosis	AFO	44.3	7762
	KAFO	24.0	4204
	Shoe Raise	11.4	2002
	FO	5.5	962
	Upper Limb	3.8	667
	SFAB	3.6	635
	Spinal	3.5	619
	Lower Limb	2.6	448
	Other	1.2	205
	Missing	0.1	19

^a^N = total number of orthoses delivered, not total number of clients.

AFO: Ankle-Foot Orthosis; FO: Foot Orthosis; KAFO: Knee-Ankle-Foot Orthosis; SFAB: Steenbeek foot abduction brace.

### Temporal trends

**Fig 2:**
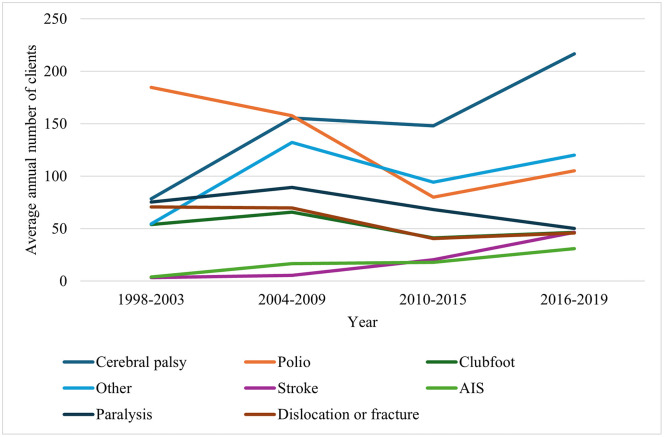
Temporal trends in reasons for orthosis use among clients across four time periods, 1998–2019. Each line represents a diagnostic category.

Across the study period (1998–2019), the average annual number of clients attending orthotic services varied substantially by diagnostic group (Fig 2). Numerical data and percentages for all graphs can be found in the supporting information (S4–S7 Tables in S1 file). Overall, several conditions demonstrated marked increases in attendance, while others showed notable declines.

The number of clients with cerebral palsy increased throughout the study period, almost doubling between 1998–2003 and 2004–2009, showing a slight dip in 2010–2015, and then rising again to the highest level in 2016–2019 (Fig 2). Stroke and AIS cases also showed continuous growth across all periods, indicating increasing demand for orthotic intervention among these populations. The ‘other’ category rose sharply between 1998–2003 and 2004–2009, with modest fluctuations thereafter.

In contrast, the number of clients with polio, paralysis, clubfoot, and dislocation or fracture generally declined over time (Fig 2). Polio and paralysis cases decreased steadily after 2004–2009, with the most marked reductions observed between 2010–2015, before levelling off in 2016–2019. Client numbers for clubfoot and dislocation or fracture also fell during the early periods and then remained relatively stable at lower levels in the later years.

**Fig 3:**
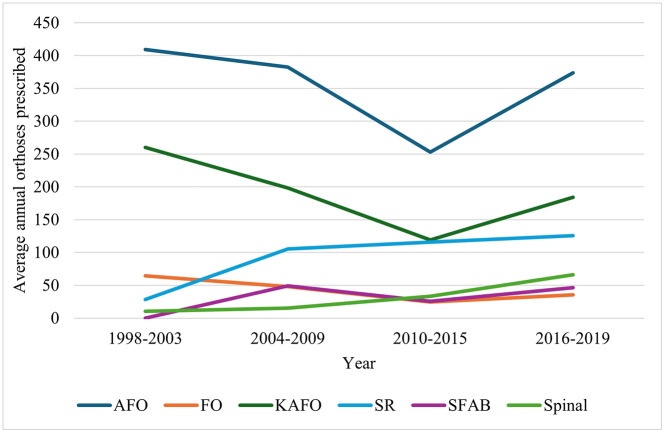
Temporal trends in orthosis device types delivered to clients across four time periods, 1998–2019. Each line represents an orthotic type category. AFO: Ankle-Foot Orthosis; FO: Foot Orthosis; KAFO: Knee-Ankle-Foot Orthosis; SR: Shoe Raise; SFAB: Steenbeek foot abduction brace.

Fig 3 illustrates changes in orthotic device prescriptions over time, highlighting the devices with the most significant variations. Fig 3 shows that AFOs and KAFOs have followed a similar prescription trend across the time periods. In 1998**–**2003, the annual average was just over 400 for AFOs and 260 for KAFOs. In 2004**–**2009, numbers declined slightly to 382 for AFOs and 198 for KAFOs, followed by a more pronounced drop in 2010**–**2015–253 and 119 respectively. In 2016–2019, there was a marked increase, with annual averages of 374 for AFOs and 184 for KAFOs respectively.

Additionally, FOs have shown a steady decline in prescriptions since 1998–2003, with a small rise in 2016–2019 that did not surpass the 1998–2003 annual rates. In contrast, SRs increased from an average of 29 per year to 125 per year by 2016–2019. Spinal orthoses also rose steadily, from 15 per year in 1998–2003–66 in 2016–2019 (Fig 3). For SFABs, numbers have fluctuated, with the 2016–2019 average (46) similar to that in 2004–2009 (49), both higher than 1998–2003 (0) and 2010–2015 (25.8) (Fig 3). The absence of cases between 1998–2003 likely reflects the fact that clinical use of SFABs did not become widespread until the mid-2000s [17].

**Fig 4:**
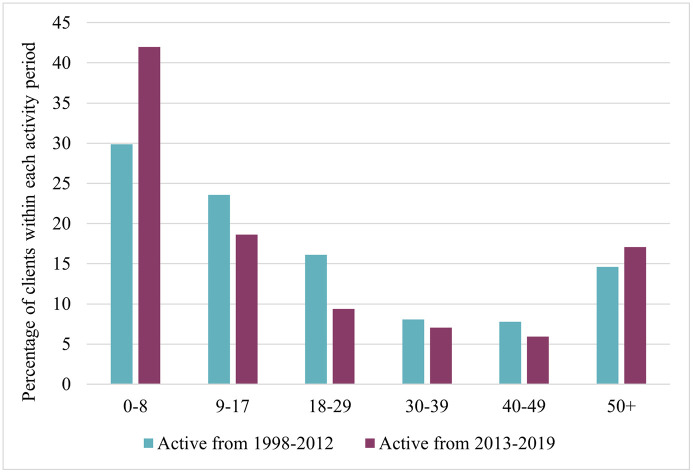
Distribution of age at first appointment for clients active before 2013 and clients active after 2013.

Fig 4 presents the age distribution of clients at their first engagement with services, grouped into two periods: 1998–2012 and 2013–2019. In the later period, a higher percentage of clients began services between ages 0–8 (42.0% vs. 29.9%), indicating a trend towards earlier intervention. In contrast, the earlier period saw greater percentages of clients initiating services at ages 9–17 (23.6% vs. 18.6%) and 18–29 (16.1% vs. 9.4%). The proportion of clients aged 50 and above increased from 14.6% in 1998–2012 to 17.1% in 2013–2019. Overall, these data suggest that services are accessed by a broader age range, encompassing both younger and older clients.

**Fig 5:**
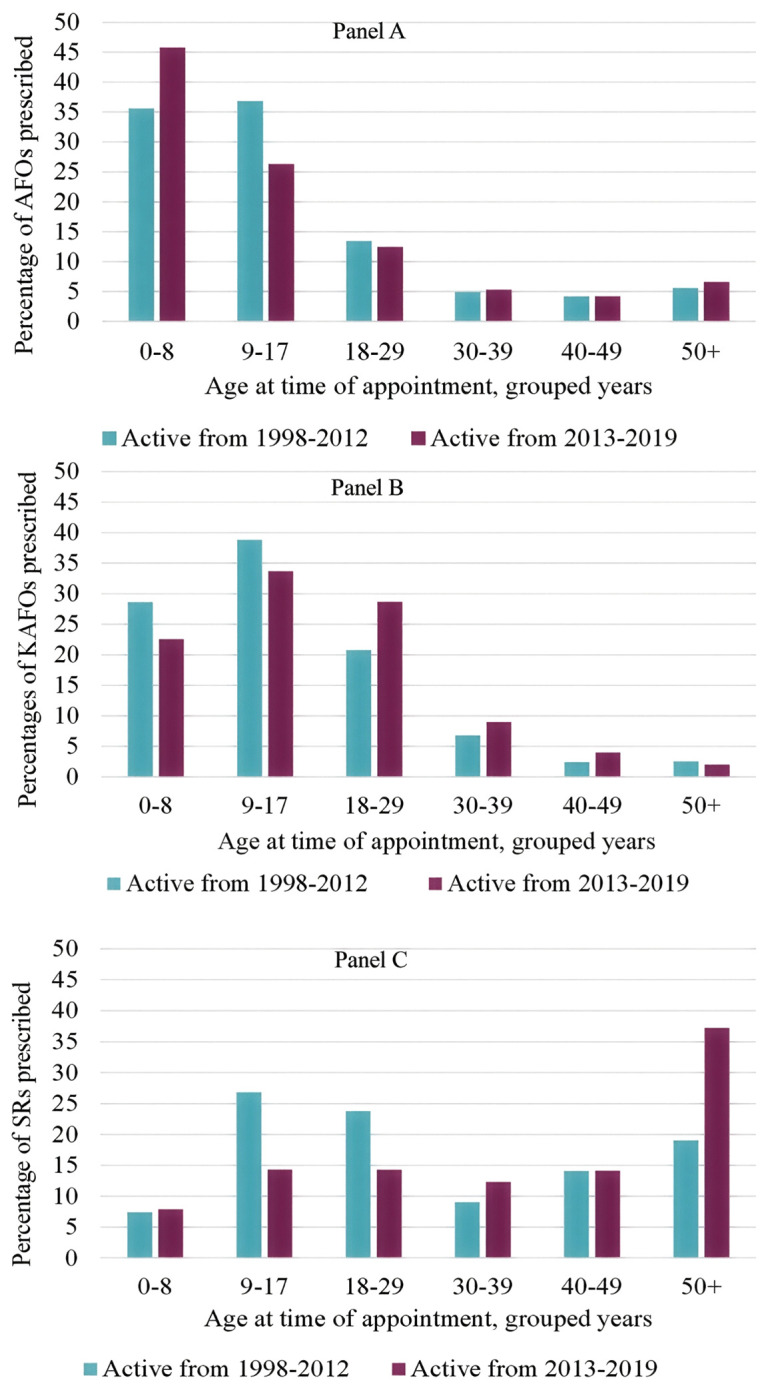
Age at time of appointment and percentage of orthoses prescribed for clients active between 1998–2012 and clients active between 2013–2019. Panel A: Percentage of AFOs prescribed to clients between 1998-2012 and 2013–2019 prescribed by grouped age at the time of appointment in years. Panel B: Percentage of KAFOs prescribed to clients between 1998-2012 and 2013-2019 prescribed by grouped age at the time of appointment in years. Panel C: Percentage of SRs prescribed to clients between 1998-2012 and 2013-2019 by grouped age at the time of appointment in years.

Changes in orthotic prescriptions between 1998–2012 and 2013–2019 are reflected in client age distributions at the time of appointment for the three most commonly prescribed orthoses (AFOs, KAFOs, and SRs, Fig 5). The differences between clients active between 1998–2012 and clients active 2013–2019 were statistically significant for all three orthotic types (AFOs: χ^2^ = 122.4, P < 0.001, KAFOs: χ^2^ = 64.1, P < 0.001, SRs: χ^2^ = 116.7, P < 0.001). The most notable shifts occurred in the youngest (0–8 years) and oldest (50 + years) age groups. For clients active in 2013–2019, 45.7% of 0–8-year-olds received AFOs, compared with 35.5% in 1998–2012, whereas in 1998–2012 the highest proportion of AFO prescriptions was among 9–17-year-olds (36.8%). Prescriptions for AFOs among adults (18–49 years) remained relatively stable across the two periods.

For KAFOs, in 1998–2012 the highest prescription rates were observed in clients aged 0–8 years (28.6%) and 9–17 years (38.9%), with percentages declining in 2013–2019; however, 9–17-year-olds remained the largest recipient group in the later period (33.7%). Prescriptions for adults aged 18–49 years increased in 2013–2019 relative to 1998–2012. SR prescriptions also shifted with age. In 1998–2012, the highest SR prescriptions were for clients aged 9–17 years (26.8%) and 18–29 years (23.8%). In 2013–2019, these proportions decreased to 14.3% for both age groups, whereas SR prescriptions for clients aged 50 + years increased from 19.0% to 37.2%, reflecting changing patterns of service use.

## Discussion

This study provides an national retrospective descriptive study of routinely collected centre management data, examining trends in orthotic service provision over two decades. The findings highlight shifts in appointment types, with repairs becoming the most common service delivered. Although appointment numbers peaked in the early 2000s, assessments have declined steadily since the mid-2000s.

Changes in client demographics suggest a growing demand for orthotic services among both younger and older populations, with cerebral palsy emerging as the most common reason for orthosis use. This pattern differs from findings reported in other contexts. For example, in the UK, paediatric orthoses represent a smaller although still significant proportion of overall orthotic orders [18]. A multi-country study of rehabilitation clinics, including in Cambodia, reported that 5.9% of users at ICRC clinics were under five years old, and 14.8% were aged 5–17 years, meaning around one-fifth of clients were under 18 [19]. This contrasts with the age distribution at Exceed clinics, likely reflecting differences in referral patterns, service models, or population needs. The study highlights the value of disaggregating data by service type, as combining all rehabilitation users obscures the higher proportion of under-18s in orthotic services.

A notable finding from this study is that Exceed has delivered nearly 50,000 orthotic appointments since 1998, with currently around 4,000 active clients. This is nearly 1,500 more than the number of active clients for prosthetics services at the same clinics (2,820 active prosthetics clients) [16]. Notwithstanding, total appointments for prosthetics were similar, suggesting that fewer prosthetic clients have more appointments per person. This likely reflects the prolonged duration of orthotic use, which results in a larger number of clients but fewer consultations per individual. Much of the existing research worldwide focuses on prosthetics, often grouping prosthetics and orthotics users together [14,20], which overlooks the distinct service needs of orthotic users. The higher number of active orthotic clients combined with the prevalence of device repair appointments and the need for long-term maintenance, highlights the need for explicit inclusion of orthotics in policy and service planning separately to other services delivered.

Despite over 4,000 active clients, the overall decline in appointment numbers may be attributable to increased access to private P&O services, reductions in polio prevalence, and improved early intervention for congenital conditions. Between 2018 and 2020, Exceed trialled a cost-recovery model, requiring clients to contribute towards new orthotic devices based on income, with economically disadvantaged individuals receiving services free of charge [21]. This policy may explain the particularly sharp decline in appointments between 2018 and 2019, as some service users were required to pay for a new orthosis, potentially discouraging them from attending or obtaining a replacement orthosis. Additionally, the introduction of a social enterprise-based ‘Modern Service Clinic’ at Exceed’s Phnom Penh site, operating separately from the free clinic, may have redirected higher-income clients away from dataset presented here. These changes were influenced by declining external support for rehabilitation services in Cambodia, necessitating alternative funding models [21]. In 2019, Exceed delivered more than 800 orthotic devices, alongside prosthetics and other mobility aids, highlighting the scale of service provision within its centres. While data from other providers such as ICRC, Humanity & Inclusion, and government-operated centres were not accessible for this study, the digital health records from Exceed offers important insight into patterns of orthotic service use in Cambodia.

Disparities in service utilisation by sex were observed, with 10% fewer female active clients than male. This is consistent with other findings that access to physical rehabilitation is impacted by gender [19,22]. The WHO-UNICEF report [2] notes that in many countries, women often have reduced access to assistive products because the services are not gender-friendly. Ensuring that female rehabilitation professionals are adequately trained is essential, as some patients may be reluctant to receive care from a provider of the opposite sex [23]. The analysis also identified significant associations between gender and reasons for orthosis use. Women and girls exhibited a higher proportion of AIS diagnoses, consistent with the established female predominance in AIS [24], whereas males exhibited a higher proportion of clubfoot diagnoses, in line with the reported male predominance in this condition [25]. These findings highlight the value of routinely analysing service utilisation by gender through digital health records to inform the planning and delivery of more equitable rehabilitation services.

Differences were observed in the ages of orthosis service users, with a trend towards greater utilisation by both younger and older clients. Consistent with previous research suggesting younger people are more likely to access rehabilitation [19], our study found increased service use among children aged 0–8 years. As children age, orthoses may require modification or replacement, creating additional demand. This highlights how orthotic needs vary substantially across age groups, depending on the condition, the stage of growth, and the therapeutic aim.

Conversely, we also observed increased use among adults over 50, indicating that demand for orthoses is not limited to younger populations and may reflect age-related changes in mobility or the need for orthosis modification in older adults. Life expectancy at birth in Cambodia increased from 58 years in 2000 [26], to 69.9 years in 2019 which may have led to an increasing number of clients over 50. Rapid economic and demographic developments have contributed to a rise in non-communicable diseases (NCDs), including diabetes, hypertension, and stroke [27,28]. These conditions, combined with a growing number of older adults requiring rehabilitation, are likely to be major factors contributing to increased orthotic service utilisation in this age group.

These trends correspond with a broader global increase in demand for physical rehabilitation services, particularly in LMICs undergoing shifts in population health and disease burden. Similarly, the increase in stroke-related orthotic prescriptions reflects the growing global burden of NCDs [29]. In many LMICs, however, the integration of NCD management within primary healthcare and the establishment of effective referral pathways to rehabilitation services remain significant challenges [3,30]. This study also found an increase in orthosis use among people with cerebral palsy, which aligns with rising prevalence trends in LMICs [31].

In Cambodia, broader developments to the health sector to increase immunisations for children, including for conditions such as polio, helped to eradicate the disease [32]. The reduction in polio cases in Cambodia since the late 1990s [33] is another factor influencing orthotic service trends. By the end of this study period, most polio-affected clients had been living with the condition for many years or experiencing post-polio syndrome rather than being newly diagnosed. Data from this study show an increase in the use of shoe raises among clients aged over 50 years. This pattern suggests the ongoing needs of ageing polio survivors, as polio remains a common cause of limb length discrepancy in older populations, explaining the higher use of shoe raises observed in this age group.

The digital health records used in this study provide detailed insight into the prevalence of different indications for orthosis use and how these patterns have changed. They highlight the varying rehabilitation and healthcare requirements across the lifespan, illustrating both the paediatric and adult orthotic needs in Cambodia. However, it is important to acknowledge the data only captures those who have accessed services and do not account for individuals who may require orthotic intervention but are unable to obtain care. Barriers to access may include financial constraints, geographic inaccessibility, limited awareness, or using alternative mobility aids such as wheelchairs [2,22]. The data from Exceed covers three provinces representing distinct zones (Tonle Sap, Phnom Penh and Coastal) [34]. Kampong Chhnang (Tonle Sap), is primarily a rural area where service users are more likely to work in informal occupations or farming [34]. In comparison, Sihanoukville (Kampong Som) is situated in the coastal ecozone, and Phnom Penh represents its own distinct zone, both reflecting different urban environments. While generalisability has limitations, this diverse geographical representation strengthens its applicability both within and beyond Cambodia.

In the absence of epidemiological and prevalence data, patient records provide real-world evidence on orthoses use [35]. While such data are often used to investigate service delivery in high-income countries, their use in LMICs remains limited due to accessibility constraints, shortages of analytical capacity, and the prioritisation of service provision over data management [36]. This study demonstrates the value of these routinely collected records for characterising orthotic service users and examining service changes in Cambodia between 1998 and 2019.

Many LMICs lack comprehensive data on assistive products and the prevalence of conditions that may benefit from orthotic intervention. The WHO-UNICEF [2] report emphasises the importance of investing in robust data systems to strengthen service quality and accessibility. This study, alongside others, [16,19,37], demonstrates the potential of administrative data to address knowledge gaps and provide essential evidence for policymakers, service providers, and international organisations. The recognised need for comprehensive, multidisciplinary documentation and continuity of care within orthotic services highlights the importance of robust digital patient record systems, such as the ICRC PMS [8,11]. As this study demonstrates, when routinely collected documentation is standardised and digitised, it can be transformed into real-world evidence to assess service delivery and guide planning, particularly in settings where formal epidemiological data are lacking. This study utilised data from the ICRC’s PMS, a widely implemented system across LMIC rehabilitation services. The analytical approach adopted here has strong potential for replication in other settings, including centres transitioning to the ICRC’s Digital Centre Management System [38].

## Limitations

Whilst there are benefits of using routinely collected data, challenges also exist. Missing and inconsistent data can limit analysis. In this study, some variables had significant gaps or had outdated information, such as the affected side of the body and client occupation, making them unusable. The ‘other’ category for reason for orthosis use was one of the largest groups. The majority of cases labelled as ‘other’ were recorded as such by Exceed, while the remainder comprised small categories of reasons that were too few in number to form separate groups. Exceed typically assigned the label ‘other’ when information was taken from medical records provided by referral centres (e.g., hospitals or health centres), where a specific diagnosis was not documented. In these cases, the data reflected exactly what was recorded in the medical notes.

Another limitation is the study’s focus on three physical rehabilitation clinics may limit generalisability to Cambodia as a whole, as the clients in Exceed clinics may not reflect the wider population. Due to the paucity of knowledge about orthotic users in Cambodia and elsewhere in lower resource settings we cannot be certain that those using the clinics are not different to elsewhere, although this is not believed to be the case. However, the ICRC system ensures uniformity in data across physical rehabilitation centres in Cambodia and other countries using the PMS-5 system. Using administrative data means this study assesses demand rather than need, so it does not account for individuals not accessing rehabilitation services [37]. Some may seek rehabilitation through private providers, or NGOs not included in this dataset, while others may receive no care. Additionally, the data lack insights into why clients become inactive, whether due to death, discontinuation of orthosis use, transition to wheelchairs, or seeking alternative P&O providers. Future research should quantify unmet need, explore access barriers, and evaluate service models for underserved populations.

## Recommendations

Despite these limitations, this study can offer several recommendations which consider the challenges faced in using the data. First, providing rehabilitation professionals with training on data entry procedures, and ensuring consistency across clinics would improve data quality and facilitate meaningful comparisons, while also supporting compliance with data protection regulations (e.g., EU General Data Protection Regulation). Second, establishing clear and consistent definitions for the reasons behind orthotic use would improve uniformity in data collection, enabling more reliable comparative analyses and contributing to more effective clinical decision-making. Third, data should include information on referral sources, such as community organisations, self-referrals, or doctor referrals. Understanding how patients learn about services can help improve outreach efforts and service accessibility.

In terms of future research, the current dataset could be further explored by integrating contextual information on broader social, political, and environmental factors that may have influenced service use patterns, such as political changes, vaccination programmes, conflict, or natural disasters. Incorporating these variables could help explain observed trends and provide a more comprehensive understanding of fluctuations in orthotic service demand over time. Additionally, future analyses could subdivide age groups in greater detail, particularly within paediatric populations, to better identify trends and needs among younger service users. This is especially important, as the findings from this study indicate that individuals under 18 years of age comprise a large proportion of orthotic service users.

## Conclusion

To date, this research is the first observational study on the demand for orthoses in Cambodia and provides important evidence to support sustained service provision. This study indicates key trends in orthotic service provision in Cambodia over two decades, showing shifts in appointment types, client demographics, and reasons for orthosis use. Repair appointments have remained stable in number over time, set against falling assessments and delivery of devices, while alongside this there is a predominance of younger clients, particularly those with cerebral palsy, reflecting changing service demands. Despite over 4,000 active clients, there has been a decline in appointments, particularly in 2019, which may indicate changes in service access or delivery post 2019 that require further exploration. The number of active orthotic clients suggest a demand for orthotic services, yet orthotics remain underrepresented in research. Dedicated orthotic-focused studies are needed to inform service development and policy. For P&O service providers, this evidence supports targeted resource allocation and sustainable service planning to meet ongoing demand.

## Supporting information

S1 File Supplementary Table 1. Total appointments and the different appointment types by year, from 1998–2019. **Supplementary Table 2.** Disaggregated reasons for orthosis use for all clients. **Supplementary Table 3.** Disaggregated orthosis types for all orthoses delivered between 1998–2019. **Supplementary Table 4.** Average number of clients per year by different reason for orthosis use. **Supplementary Table 5.** Average number of orthoses delivered by type of orthoses for all clients. **Supplementary Table 6.** Percentage of clients at age of first appointment by whether they were active between 1998-2012 or active from 2013-2019. **Supplementary Table 7**. Change over time in orthoses delivered by age at time of orthosis delivery for clients active between 1998-2012 or active from 2013-2019, percentages.
(ZIP)
