# Supplementary material for: Examining the changing demand for orthotic service provision from routinely collected digital patient data: A national retrospective descriptive study across three clinics in Cambodia
Source: PLoS One. 2025 Dec 9;20(12):e0338461. doi: 10.1371/journal.pone.0338461 (PMC12688099; doi:10.1371/journal.pone.0338461)
Supplement: S1 File — Supplementary Table 1. Total appointments and the different appointment types by year, from 1998–2019. Supplementary Table 2. Disaggregated reasons for orthosis use for all clients. Supplementary Table 3. Disaggregated orthosis types for all orthoses delivered between 1998–2019. Supplementary Table 4. Average number of clients per year by different reason for orthosis use. Supplementary Table 5. Average number of orthoses delivered by type of orthoses for all clients. Supplementary Table 6. Percentage of clients at age of first appointment by whether they were active between 1998-2012 or active from 2013-2019. Supplementary Table 7. Change over time in orthoses delivered by age at time of orthosis delivery for clients active between 1998-2012 or active from 2013-2019, percentages. (ZIP) [file pone.0338461.s001.zip › S5 File .docx]

**S5 File.**

**Supplementary Table 5.** Average number of orthoses delivered by type of orthoses for all clients.

|  | **Year** | | | |
| --- | --- | --- | --- | --- |
| **Type of orthoses** | **1998-2003** | **2004-2009** | **2010-2015** | **2016-2019** |
| AFO | 409.2 | 382.3 | 253.2 | 373.5 |
| FO | 64.3 | 48.0 | 24.5 | 35.3 |
| KAFO | 260.3 | 198.2 | 119.3 | 184.3 |
| SR | 28.5 | 105.5 | 115.8 | 125.8 |
| SFAB | 0.2 | 49.0 | 25.8 | 46.3 |
| Spinal | 10.3 | 15.3 | 33.5 | 66.0 |
| Upper limb | 21.3 | 50.5 | 21.2 | 27.3 |
| Lower limb | 41.2 | 12.3 | 8.3 | 19.3 |
| Other | 7.5 | 19.2 | 3.8 | 5.5 |
| Missing | 0.2 | 0.0 | 1.0 | 3.0 |
